# The atmosphere: a transport medium or an active microbial ecosystem?

**DOI:** 10.1093/ismejo/wrae092

**Published:** 2024-05-28

**Authors:** Rachael Lappan, Jordan Thakar, Laura Molares Moncayo, Alexi Besser, James A Bradley, Jacqueline Goordial, Elizabeth Trembath-Reichert, Chris Greening

**Affiliations:** Department of Microbiology, Biomedicine Discovery Institute, Monash University, Clayton, Victoria 3800, Australia; School of Earth, Atmosphere & Environment, Monash University, Clayton, Victoria 3800, Australia; Securing Antarctica’s Environmental Future, Monash University, Clayton, Victoria 3800, Australia; School of Environmental Sciences, University of Guelph, Guelph, Ontario N1G2W1, Canada; School of Geography, Queen Mary University of London, London E1 4NS, United Kingdom; Natural History Museum, London SW7 5BD, United Kingdom; Aix Marseille University, University of Toulon, CNRS, IRD, MIO, Marseille 13009, France; School of Earth and Space Exploration, Arizona State University, Tempe, AZ 85287, United States; Aix Marseille University, University of Toulon, CNRS, IRD, MIO, Marseille 13009, France; School of Biological and Behavioural Sciences, Queen Mary University of London, London E1 4NS, United Kingdom; School of Environmental Sciences, University of Guelph, Guelph, Ontario N1G2W1, Canada; School of Earth and Space Exploration, Arizona State University, Tempe, AZ 85287, United States; Department of Microbiology, Biomedicine Discovery Institute, Monash University, Clayton, Victoria 3800, Australia; Securing Antarctica’s Environmental Future, Monash University, Clayton, Victoria 3800, Australia

**Keywords:** Atmospheric microbiology, aeromicrobiology, bioaerosols, low-biomass, microbial ecology, metagenomics, metabolic activity

## Abstract

The atmosphere may be Earth’s largest microbial ecosystem. It is connected to all of Earth’s surface ecosystems and plays an important role in microbial dispersal on local to global scales. Despite this grand scale, surprisingly little is understood about the atmosphere itself as a habitat. A key question remains unresolved: does the atmosphere simply transport microorganisms from one location to another, or does it harbour adapted, resident, and active microbial communities that overcome the physiological stressors and selection pressures the atmosphere poses to life? Advances in extreme microbiology and astrobiology continue to push our understanding of the limits of life towards ever greater extremes of temperature, pressure, salinity, irradiance, pH, and water availability. Earth’s atmosphere stands as a challenging, but potentially surmountable, extreme environment to harbour living, active, resident microorganisms. Here, we confront the current understanding of the atmosphere as a microbial habitat, highlighting key advances and limitations. We pose major ecological and mechanistic questions about microbial life in the atmosphere that remain unresolved and frame the problems and technical pitfalls that have largely hindered recent developments in this space, providing evidence-based insights to drive future research in this field. New innovations supported by rigorous technical standards are needed to enable progress in understanding atmospheric microorganisms and their influence on global processes of weather, climate, nutrient cycling, biodiversity, and microbial connectivity, especially in the context of rapid global change.

## An ocean of air: the significance of the atmosphere as a microbial ecosystem

In 1644, the physicist Evangelista Torricelli wrote a letter that contained the phrase “Noi viviamo sommersi nel fondo d’un pelago d’aria,” or “We live submerged at the bottom of an ocean of air” [1]. Torricelli was addressing the significance of barometric pressure, but this “ocean of air” is an apt concept for our consideration of the atmosphere as a microbial habitat, as presented by Womack *et al.* in 2010 [2]. The atmosphere forms a blanket over the lithosphere, hydrosphere, and cryosphere, which are well-known microbial ecosystems. It is reasonable that the atmosphere is another planetary scale habitat harbouring microbial communities that interact with their environment and exert significant influence over global-scale processes, yet few have considered the possibility prior to the recent renewed interest in atmospheric microbial ecology [3–7]. A recent estimate of the biomass distribution on Earth omits atmospheric biomass [8], reflective of frequent lack of consideration of the atmosphere as part of the biosphere. Yet, broad estimates of cell abundance in the atmosphere suggest that near-surface air contains 10^4^ to 10^5^ microbial cells per m^3^ [9], scaling to a total of 5 × 10^22^ cells in the troposphere [10]. While these estimates contain substantial variability across regions and quantification approaches, the atmosphere is nonetheless a substantial habitat about which we understand disproportionately little despite its planet-wide coverage.

The atmosphere’s most evident ecological role is as a transport medium. Dispersal of microorganisms through the air is a critical ecological process in the spread of pathogens over short (e.g. SARS-CoV-2, measles [11]) and long (e.g. fungal spores responsible for wheat rust affecting agriculture [12]) distances. The atmosphere also plays a role in the evolution and maintenance of global biodiversity [13], with suggested evidence of intercontinental transmission of bacteria [5, 14, 15], which are estimated to reside in the atmosphere for 2–15 days [16]. Lourens Baas-Becking’s oft-used quote “everything is everywhere, but the environment selects” indeed originally refers to the atmosphere’s presumed homogeneous distribution of microorganisms across habitats and suggests that those organisms most adapted to a given surface environment would survive upon deposition and reside in a suitable niche [17]. However, this hypothesis has been frequently challenged by the recognition that it is simply not possible for all extant microorganisms to concurrently be everywhere in the atmosphere, and evidence indeed suggests that microorganisms are dispersal limited [18, 19]. In turn, this places the atmosphere not as a reservoir of all microorganisms on the planet, but as an intermediate habitat containing microbial communities constrained by inputs from surface environments, survival and residence times, and other physicochemical factors.

Classically, the atmosphere has been viewed as a conduit for the passive dispersal of microorganisms via random (i.e. neutral) processes [20]. Under this model, suspended microorganisms may be largely inactive during their transit before eventual deposition in a geographically distinct and potentially more suitable habitat [21]. However, the atmosphere is a hostile environment requiring resilience to freezing temperatures, high ultraviolet (UV) radiation, oxygen radicals, and desiccation [21], suggesting selection likely occurs prior to deposition (i.e. niche processes). If the atmosphere were instead considered to be a “true” ecosystem, by definition, this would comprise metabolically active organisms interacting with their environment and each other while suspended in the air [2, 4]. Such an atmospheric ecosystem may comprise multiple distinct habitats (e.g. dust, clouds, methane-rich air) and host resident microorganisms that could profoundly influence global biology, chemistry, and climate beyond the current paradigm [2, 5, 13].

Microbial life on Earth is diverse and resilient, with survival capabilities that regularly push the boundaries of previously established physicochemical limits of life. Psychrophilic microorganisms can divide at temperatures as low as −15°C (*Planococcus halocryophilus)* [22] and metabolize at much lower temperatures [23, 24]; deeply buried microorganisms can survive at extraordinarily low rates of energy use [25]; polyextremophiles can resist multiple stressors including desiccation and ionizing radiation (e.g. *Deinococcus radiodurans*) [26]; and notably, microorganisms can metabolize atmospheric concentrations of trace gases (e.g. H_2_, CO, CH_4_) to support their energetic needs [27–30]. Life in the atmosphere is certainly challenging, but the conditions experienced by atmosphere-dwelling microbial cells are well within the boundaries currently considered to limit life. Indeed, exchange with physiologically challenging surface environments (e.g. the Atacama Desert, Antarctic dry valleys, or the cryosphere) may seed the atmosphere with resilient microorganisms that are well suited to atmospheric survival.

**Figure 1:**
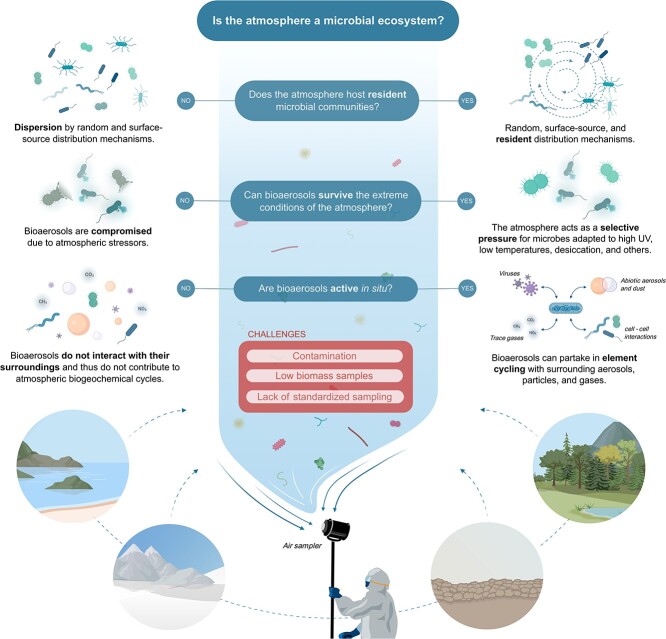
Null and alternative hypotheses on significant fundamental questions and their implications for atmospheric microbial ecology. Created with BioRender.com.

A critical question in aerobiology remains unresolved: does the atmosphere support microbial life only through passive dispersal, or does it additionally host an active resident microbiome that interacts with other ecosystem components? We foresee a plethora of research opportunities to fill knowledge gaps in atmospheric biodiversity, microbial activity, and adaptation to environmental stresses (Box 1, Fig. 1), akin to those that have driven decades of research in terrestrial and aquatic ecosystems.

Box 1. Key open questions in atmospheric microbiology

1. *Does the atmosphere contribute only to the dispersal of microorganisms, or does it additionally host a resident microbial community shaped by selection pressures?*

This fundamental question has been posed several times in recent years [2, 4–6]. Despite decades of research, it has not been empirically determined whether the atmosphere hosts resident (either permanent or temporary) microbial communities, as other environmental compartments on the planet do. Are atmospheric microbial communities assembled via stochastic, random aerosolization and subsequent mixing of terrestrial and marine microorganisms, or do environmental conditions and other factors select for organisms that are more readily aerosolized and can survive in the atmosphere? Is the atmospheric microbiome shaped by selection of these adapted microorganisms, or by “selective death” of organisms that cannot survive atmospheric conditions? To what extent is microbial survivability shaped by chance rather than specific adaptations? Is there a continuous, resident microbiome in the atmosphere, or is microbial community composition dynamic, with regular turnover of organisms via mixing with adjacent environments that vary across geographical locations? The concept of obligately atmospheric, or “Peter Pan” (never landing) microorganisms has recently been proposed [3] and presents a curious avenue of study that will help to define the nature of the atmospheric ecosystem and the requirements to sustain atmospheric microbial life.

2. *How do atmospheric microorganisms persist in the extreme but likely habitable conditions in the atmosphere?*

The atmosphere poses harsh conditions to life, including desiccation, exposure to UV radiation, low temperature, and low pressure. What molecular or physiological mechanisms do microorganisms employ to survive these conditions? Is this a matter of tolerance and stress resilience until deposition in a suitable environment, or do some atmospheric organisms prefer such extremes? Is there a combination of challenging conditions in the atmosphere that precludes long-term survival of life [31–33]? Does life in the atmosphere consist only of spores and cells that are inactive until deposited, or of cells that are both active and resilient? Is survival sustained by particle association, which may provide some nutrients, protection, or attract water vapour?

3. *Are microorganisms active and carrying out functional ecosystem roles in the atmosphere?*

Preliminary evidence suggests that certain microorganisms are viable and actively metabolizing in the atmosphere [34–38], though this is not well demonstrated outside of *in vitro* experiments. What roles do atmospheric microorganisms play in the wider ecosystem, and potentially in global atmospheric processes? How does atmospheric residence time impact microbial activity, and does activity change with time through loss of viability or stimulation by particular microenvironments (e.g. clouds)? It is feasible that atmospheric microorganisms could contribute to global biogeochemical processes through metabolic activities, including carbon fixation or degradation of carbon compounds (e.g. pollutants); enhancing or competing with abiotic reactions; or by the production or consumption of climate-relevant gases, an ecological role and metabolic strategy with increasingly recognized importance in other low biomass microbial ecosystems such as arid deserts and the cryosphere [30, 39].

Additionally, atmospheric microorganisms likely influence global biodiversity through dispersal and exchange of organisms between environments, including the transmission of pathogens, antimicrobial resistance genes, mobile genetic elements, and phages. Low levels of biomass in the atmosphere may correspond to low microbial activity, but is this collectively important on a global scale? How significant could this contribution be to global phenomena? Interdisciplinary efforts are essential for both experimental and detailed modelling studies to understand the role of the atmosphere in biogeochemical cycling.

4. *What is the impact of climate change on atmosphere-dwelling microorganisms and their potential role in biogeochemical cycling?*

If atmospheric microorganisms contribute to regional and global element cycling, climate, and atmospheric processes, as is the case with soil and marine microorganisms, it is reasonable to assume an interplay between climate change and these activities. How will warming, increased extreme weather events, increasingly ice-free areas, and changes in atmospheric gas and aerosol composition influence atmospheric microbial communities and their activities?

## Are atmospheric microbial communities structured and adapted?

Addressing the composition, structure, and adaptations of an entire atmospheric microbial community is a task well suited to comprehensive molecular approaches. Molecular tools have enabled many insights into atmospheric microbial ecology, as they are highly sensitive and capable of detecting organisms from multiple domains of life. Recent ribosomal amplicon sequencing studies have consistently revealed that airborne microbiota are primarily a collection of organisms sourced from terrestrial and marine inputs, with increased representation of “local” microorganisms from underlying or adjacent environments [14, 15, 40–43]. Airborne microbial assemblages can be highly variable, fluctuating daily and seasonally [42, 44–47], differing with biogeography [40, 43], and have been observed to “wash out” of the atmosphere with precipitation events [48]. These findings imply that the atmospheric microbiome is a “moving imprint” of surface ecosystems (aptly described by Amato *et al.* [49]), rather than a community of organisms endemic only to the atmosphere. Reciprocally, the atmosphere contributes new microorganisms to terrestrial ecosystems via dispersion, deposition, and subsequent colonization [50–52]. This transient nature of the atmospheric microbiota makes for a uniquely dynamic ecosystem, but does not preclude active or long-term resident populations, nor a structured community shaped by selection pressures, which remain poorly explored. A key component here is whether the communities exhibit evidence of selection and adaptation.

Generally, ribosomal amplicon sequencing approaches targeting prokaryotic or fungal marker genes are robust to contaminating nonmicrobial DNA and remain a widely used and powerful technique for biodiversity profiling. However, the primers, sequencing platforms, clustering methods, and reference databases used to amplify, sequence, and analyse marker genes of interest introduce varied taxonomic biases [53]; thus, comparisons and syntheses of aerobiological amplicon datasets should take such effects into account. Standardized primer sets and protocols have been recommended for other environments (e.g. The Earth Microbiome Project [54]), but the specific primers and bioinformatics pipelines aerobiology researchers select will depend on their research questions and study sites and must include careful consideration of bias against particular taxonomic groups. Further, amplicon sequencing provides only taxonomic information at sometimes limited resolution. Consequently, much of the atmospheric microbial ecology literature has focused on community composition over traits and do not provide sufficient information to address key questions about atmospheric microbial activities, persistence, sustenance, adaptation, and survival (Table 1).

**Table 1:** Overview of critical concepts regarding the atmosphere as a microbial ecosystem and their associated level of understanding, evidence, and obstacles.

What is understood about…	Level of understanding	What is the evidence?	What are the obstacles to our understanding?
1. The presence of microorganisms in the atmosphere	High understanding	Cultured isolates from aerosol samples [35, 37, 38, 55, 56]. Molecular work (DNA and RNA detection) Reviewed in [4]. Intact cells visualized with microscopy [38, 57–59].	Low biomass samples. Contamination
2. The role of the atmosphere as a dispersal mechanism only, or additionally as a habitat for a resident microbial community shaped by selection pressures	Low–medium understanding	Molecular work (mostly 16S rRNA and internal transcribed spacer (ITS) amplicon sequencing) confirms atmospheric distribution is strongly tied to surface sources [14, 15, 40–43].	Limited sample size. Low biomass samples. Contamination Lack of standardized sampling methodology. Poorly standardized cataloguing of environmental conditions from sampling locations.
3. The viability of microorganisms in the atmosphere	Low–medium understanding	Culture-based growth confirms the presence of viable bioaerosols [35, 37, 55, 56]. Viability staining confirms the presence of viable cells after artificial aerosolization [60]. Fluorescence microscopy confirms that bioaerosols can have intact membranes after aerosolization [61].	Low biomass samples. Contamination Lack of standardized sampling methodology. Difficulty mimicking atmospheric environmental conditions. Lack of outdoor field studies. Poorly standardized cataloguing of environmental conditions from sampling locations.
4. The *in situ* activity of atmospheric microorganisms	Low understanding	Substrate-incubation of artificially aerosolized cells shows bioaerosols can metabolize while suspended [62–64]. Molecular work (metatranscriptomics) identifies potentially active taxa that contain relatively abundant RNA [65–67]. Fluorescent activity stains show that bioaerosols can stay active after aerosolization [61].	Low biomass samples. Contamination Lack of standardized sampling methodology. Sampler interference on cell activity. Poorly standardized cataloguing of environmental conditions from sampling locations. Deficiency of activity measurements on outdoor field samples. Unknown effects of residence time on survival and activity
5. The contribution of atmospheric microorganisms to atmospheric biogeochemical cycles	Low to none	No clear evidence	Same as question 4.
6. The influence of climate change on atmosphere-dwelling microorganisms and their potential role in biogeochemical cycling	Low to none	No clear evidence	Low biomass samples. Contamination Lack of standardized sampling methodology. Need for longer-term and larger-scale sampling campaigns. Poor understanding of potential atmospheric microbial ecosystems.

Untargeted shotgun metagenomics enables the assembly of microbial genomes of both known and unknown organisms. It provides genome-wide information about bacterial, archaeal, eukaryotic, and viral taxonomic diversity and metabolic capabilities, including a genetic basis for survival mechanisms. Though this approach has been advocated for use in atmospheric microbiology for some time [68], few studies have attempted metagenomics on atmospheric samples. Two dust and particulate matter studies have indicated that dust-associated communities harbour certain enriched genes, including UV-induced DNA damage repair, sporulation [69], and degradation of organic contaminants (e.g. benzoate and aminobenzoate) [70] compared to communities from the surrounding environment. These findings suggest adaptation to atmospheric conditions and imply that anthropogenic factors impact the atmospheric microbiome. Tignat-Perrier *et al.* sought to identify an atmosphere-specific functional signature by examining nine sites of different elevation and environment type [71]. While they discovered that stress response traits (sporulation, response to UV, oxidative stress, and desiccation) were, on average, more abundant in air compared to surrounding soil, seawater, or snow samples, there were no functions determined to be specific to atmospheric communities [71]. These aforementioned stress-tolerance genes largely corresponded to fungi, which were dominant in these air samples, leading the authors to speculate that fungi may survive atmospheric conditions better than bacteria [71]. There is a notable absence of atmospheric (especially nonurban) metagenomes in the literature. Furthermore, several additional studies that have generated metagenomic data have primarily reported taxonomic patterns, with no observations on functional capabilities [72–76]. Quality atmospheric metagenomics studies have the potential to fill the substantial knowledge voids outlined in Box 1, though it is vital to combine this with other methods and approaches. For example, it is unknown whether the abundance of traits such as sporulation and UV tolerance reflect an atmospheric lifestyle or an enrichment of contaminating organisms that are resistant to decontamination procedures. This a significant barrier to interpretation of atmospheric metagenomic studies that must be carefully and systematically addressed.

Metagenomic analysis of the atmosphere represents an exciting advance in quantifying atmospheric biodiversity and potential metabolic functions. However, the sensitive technique is a double-edged sword: molecular studies (both amplicon and metagenomic) of atmospheric microorganisms can be severely impacted by contaminating DNA [74, 76–80], the importance of which has been recognized as remarkably overlooked, dismissed, or minimally discussed in most studies [81, 82]. Cells or DNA originating from the sampling and handling personnel, equipment, laboratory reagents, or laboratory air can collectively contribute to contamination [77, 81], which is often a negligible issue for high-biomass samples but becomes increasingly confounding with low-biomass samples. Contamination in microbiome studies is a well-documented challenge that requires a high degree of care in sample collection and preparation (e.g. extensive protective personal equipment (PPE), decontamination, and clean laboratory practices), in combination with sufficient and relevant negative controls and transparent reporting of these procedures and their effectiveness [82–86]. When contamination controls are not described, it is very challenging to interpret how much or which aspects of a study’s findings relate to genuine biological signals, which, in turn, risks misinterpretation and inaccurate findings permeating the field. Importantly, combining molecular techniques with other methods such as microscopy, biogeochemical assays, or culture-based approaches is critical to validate and extend molecular findings and to expand our knowledge on microbial viability and activity in the atmosphere.

## Evidence of activity in the largest but least explored microbial ecosystem

To begin to understand the potential ecosystem roles of atmospheric microorganisms, it is critical to examine microbial viability and activity in conjunction with recent insights on community structure offered by molecular approaches. Traditional aerobiology research has focused largely on the study of airborne pollen and fungal spores, and despite recent molecular advances in the field, evidence surrounding the activities and functions of atmospheric microorganisms is scarce (Table 1). Furthermore, there is a bias towards studying indoor air microbiomes relative to outdoor environments that limits our ecosystem-level knowledge of bioaerosols [87]. However, both early and more recent culture-based studies are providing a growing, though still limited, body of evidence for the metabolic activity of microorganisms in the atmosphere. Atmospheric microbiology began with 19th-century experiments by Pasteur, Tyndall, and others showing that viable microorganisms could be grown from the air [88]. In 1975, Dimmick *et al.*’s atomizer experiment suggested that aerosolized *Serratia marcescens* were capable of metabolizing glucose while suspended in the air, at 90%–95% relative humidity and 21°C [62]. This was seemingly the first study to establish that bacteria are not inactive when aerosolized, at least over short timescales. Similar studies have demonstrated low-affinity (1500 ppmv) methane oxidation by cells aerosolised from mixed cultures, as detected by ^13^C incorporation into DNA following incubation with ^13^CH_4_, [63] as well as volatile organic compound degradation by *Sphingomonas aerolata*, as observed indirectly by total cellular ribosomal RNA (rRNA) content [64]. Importantly, the survival and activity of aerosolised cultures depend substantially on the aerosolization technique due to its impact on membrane integrity [61].

Ice-nucleating bacteria such as the plant pathogen *Pseudomonas syringae* reside within clouds and have been known since the 1980s to directly contribute to their formation, a mechanism now widely accepted as a major driver of cloud formation [89–93]. However, organic particles, including bacteria without ice-nucleating capabilities and nonliving cells, can act as cloud condensation nuclei [34, 36, 89, 93], so it remains unresolved whether cloud formation is an active process mediated by the bacterium and whether bacteria-mediated cloud formation is substantial at a global scale. Multiple laboratory experiments suggest that microbial activity is sustained within clouds. Cloud-derived isolates degrade or transform organic compounds [35, 37, 55, 56] and produce adenosine triphosphate (ATP) [94]. One study to date has combined metagenomics with metatranscriptomics to explore potential microbial activities in clouds, reporting transcription of genes associated with oxidative stress tolerance mechanisms and metabolism of one-carbon compounds [65].

Outside the cloud habitat, some studies have directly detected cellular ATP in atmospheric samples, inferring the quantity of viable cells [38] and relative level of activity [95]. However, these measurements may fail to capture cellular ATP in spores and provide only a broad sample-wide estimate of total or bulk activity. Viability and vitality stains, in combination with fluorescence microscopy and fluorescence activated cell sorting (FACS), have also been used to quantify active or viable bioaerosols [60, 61, 96]. RNA-based ribosomal amplicon approaches have provided some molecular evidence for microbial activity and viability in atmosphere-dwelling communities, enabling a comparison of the “total” (DNA) and “active” (RNA) community fractions. This approach can highlight active taxa that are rare in the DNA-based community profile [66, 67], but require careful interpretation of “phantom” taxa that appear only in the active fraction.

While these important advances begin to address the survivability and potential functions of atmospheric bacteria, our current knowledge is largely based on indirect *in vitro* evidence of bacterial atmospheric activities and largely addresses their ability to become active once incubated under controlled conditions, rather than providing evidence for activity in their atmospheric habitat. Factors such as the type of sampling (liquid-based or dry), and the sample storage and processing conditions will impact measured cell activity. Furthermore, *in situ* conditions (e.g. humidity, wind speed, air temperature, UV) are poorly standardized and seldom collected continuously throughout sampling [97], and there is great need for community consensus in the minimum set of metadata that should accompany atmospheric samples. It remains challenging to reproduce the environment and potential interactions experienced by atmospheric microorganisms in the laboratory; thus, most studies have relied on culturable representatives of the atmospheric microbial community. This leaves an important gap in knowledge: like most other environmental microbiomes, a substantial portion of the atmospheric microbiome likely includes viable but nonculturable cells that are overlooked with common *in vitro* methods [98, 99].

## Considerations for effectively capturing microorganisms from the atmosphere

Regardless of the research focus and downstream approach, characterizing accurate microbiological and ecological patterns from bioaerosols requires collection of sufficient biomass from the air: this is heavily influenced by sampling methodology, which remains poorly standardized in the field [78, 100]. Existing sampling technologies present unique contamination risks along the pipeline from collection to analysis, differing capacities to preserve cell vitality and viability [97, 101], and size distribution biases for captured bioaerosols [102]. No single protocol is sufficient for all research objectives that may have different priorities for sample quality (e.g. DNA sequencing compared to activity measurements), resulting in uncomfortable trade-offs between sampling rate, capture efficiency, storage medium, and importantly, financial cost: prompting smaller sample sizes and reduced power in an environment that is already sparse and highly stochastic in nature. Aerobiology sampling technologies are compared and reviewed in depth elsewhere in the literature [97, 102–104]; however, protocol optimization for outdoor bioaerosol samples, particularly in the context of measuring cell activity, remains poorly developed [87, 105]. Aerobiology researchers should clearly detail sampling equipment and methods [97], informing robust benchmarking studies to improve inter-study comparability and expedite methodological innovations. Sampling parameters will have significant impacts on cellular properties measured by downstream analyses, and ultimately our capacity to answer fundamental questions (Box 1) where informed, tailored methodological choices must be made with available technologies.

## Summary

There remain many unknowns about life in our ocean of air. Our conception of the atmosphere as an active microbial ecosystem will lead to significant advances in our understanding of one of Earth’s largest but least explored ecosystems, how it interacts with other major ecosystems on the planet, and how it contributes to global biogeochemical cycling and atmospheric processes. By increasing research efforts with greater temporal and spatial assessments of the atmosphere combined with stringent approaches, this new knowledge will assist us in predicting interactions between a changing climate and atmospheric life and provide valuable insights on the potential survivability of life on “thin air.”

## Data Availability

Data sharing not applicable to this article as no datasets were generated or analysed during the current study.
